# Reduced plant competition among kin can be explained by Jensen's inequality

**DOI:** 10.1002/ece3.1312

**Published:** 2014-11-10

**Authors:** Anna K Simonsen, Theresa Chow, John R Stinchcombe

**Affiliations:** 1 Department of Ecology and Evolutionary Biology, University of TorontoToronto, Ontario, Canada; 2 Department of Ecology and Evolutionary Biology & Centre for the Analysis of Genome Evolution and Function, University of TorontoToronto, Ontario, Canada

**Keywords:** Jensen's inequality, kin competition, kin recognition, niche partitioning, plant–plant interactions, rhizobia, sibling, tragedy of the commons

## Abstract

Plants often compete with closely related individuals due to limited dispersal, leading to two commonly invoked predictions on competitive outcomes. Kin selection, from evolutionary theory, predicts that competition between relatives will likely be weaker. The niche partitioning hypothesis, from ecological theory, predicts that competition between close relatives will likely be stronger. We tested for evidence consistent with either of these predictions by growing an annual legume in kin and nonkin groups in the greenhouse. We grew plant groups in treatments of symbiotic nitrogen fixing bacteria differing in strain identity and composition to determine if differences in the microbial environment can facilitate or obscure plant competition patterns consistent with kin selection or niche partitioning. Nonkin groups had lower fitness than expected, based on fitness estimates of the same genotypes grown among kin. Higher fitness among kin groups was observed in mixtures of N-fixing bacteria strains compared to single inoculations of bacteria strains present in the soil, which increased fitness differences between kin and nonkin groups. Lower fitness in nonkin groups was likely caused by increased competitive asymmetry in nonkin groups due to genetic differences in plant size combined with saturating relationships with plant size and fitness- i.e. Jensen's inequality. Our study suggests that microbial soil symbionts alter competitive dynamics among kin and nonkin. Our study also suggests that kin groups can have higher fitness, as predicted by kin selection theory, through a commonly heritable trait (plant size), without requiring kin recognition mechanisms.

## Introduction

Plant fitness is strongly determined by the presence or absence of competing neighbors (Harper 1977). Because seed and pollen dispersal is often limited, plants will tend to interact more frequently with closely related neighbors (Levin 1988), leading to a fundamental and largely unresolved question: What are the ecological and evolutionary consequences of competing with closely related kin (File et al. 2012b)? In populations that exhibit strong spatial genetic structure, the total fitness of an individual within a group can also be determined by fitness of its’ neighboring relatives, that is, its inclusive fitness (Hamilton 1964). If selection favors traits that reduce local competition between close relatives, competition among siblings is expected to be weaker than competition between nonrelatives (Platt and Bever 2009; File et al. 2012b). Competition between plants, in particular, could be altered or modified by soil microbes (Van der Putten and Peters 1997; Reynolds et al. 2003); however, few studies have examined the potential for microbes to modify competition between related individuals (File et al. 2012a). Here, we evaluate the consequence of competition between kin and nonkin on fitness and determine whether symbiotic soil microbes alter competitive outcomes in kin and nonkin environments.

Evidence that competition is lower in kin plant groups is consistent with the explanation that selection reduces negative competitive interactions among related individuals in a group (Willson et al. 1987; Tonsor 1989; Donohue 2003). However, the mechanisms that reduce local competition between neighboring plants remain intensely debated. Empirical studies that compete siblings and nonsiblings together in mixed sibling groups have found that several plant species can alter competition-related traits, such as root–shoot biomass allocation, depending on whether their neighbors are kin or nonkin (e.g., Dudley and File 2007; Murphy and Dudley 2009; Bhatt et al. 2011; Biernaskie 2011). Plasticity in root to shoot allocation has been interpreted as a plant's ability to recognize kin, which increases inclusive fitness by reducing energetic costs that would otherwise be allocated to exploitative or interference competition (e.g., Dudley and File 2007; Murphy and Dudley 2009). Another mechanism that may cause lower fitness in nonkin groups is the presence of higher asymmetric competition, due to genetic differences among individuals in either overall size or competitive ability, which has been suggested by authors studying *Cakile edentula* (Donohue 2003) and *Plantago lanceolata* (Tonsor 1989). Groups comprised of less related individuals will have higher asymmetry in size or competitive ability simply due to the expression of quantitative genetic variation in these traits (Tonsor 1989; Masclaux et al. 2010). Accordingly, higher variance in size or competitive ability, combined with saturating nonlinear relationships between size or competitive ability and fitness, will depress fitness in nonkin groups as a mathematical expectation through Jensen's inequality (Jensen 1906; Łomnicki and Symonides 1990).

Alternatively, fitness could be higher in nonkin groups, and a commonly proposed explanation for this phenomenon is ecological niche partitioning (Barton and Post 1986). In ecological niche partitioning, reduction in the degree of niche overlap reduces competition by maximizing resource use, thus maximizing fitness among all individuals within the group (i.e., resource complementarity; c.f. Tilman et al. 1997). Evidence consistent with the niche-partitioning hypothesis has been reported in several studies measuring the effects of kin and nonkin competition on fitness or a fitness proxy (i.e., biomass) (e.g., Escarré et al. 1994; Cheplick and Kane 2004; Boyden et al. 2008; Milla et al. 2009). Furthermore, genotypic variation in optimal environmental growing conditions is commonly found in plants (e.g., Garbutt et al. 1985; Hughes et al. 2009; Chang and Smith 2013), which can potentially maximize total group productivity (e.g., Hughes and Stachowicz 2004; Crutsinger et al. 2006; Johnson et al. 2006), provided there is sufficient environmental heterogeneity at the patch or group scale. However, it is unclear what biological mechanisms would produce nonoverlapping niches among unrelated individuals (Cheplick 1992), especially if environmental conditions acting within a group are homogenous, which would predict higher fitness among only a subset of favored genotypes in the group due to competitive exclusion.

Soil microbes could potentially play a large role in influencing the outcome of competitive interactions among kin and nonkin. The variance in individual performance of particular genotypes may be due to the identity or community composition of microbial strains (Heath and Tiffin 2007). Context-dependent performance of plant genotypes driven by microbial strain identity has been found between host plants and mutualist and pathogenic microbial symbionts (Parker 1995; Salvaudon et al. 2005; Heath et al. 2010). Thus, one potential mechanism where niche partitioning may occur between plant genotypes is through differential interaction with soil microbes (Bever et al. 2010). For example, if a particular mutualistic strain can provide larger fitness benefits to a particular plant genotypes (e.g., G × G interactions; see Parker 1995; Heath et al. 2010), some genotypes may be able to specialize in resource acquisition by forming more specialized associations with particular subsets of strains in the soil (Wilkinson and Parker 1996). Thus, variation in soil microbial strains could provide a biotic mechanism that maintains genetic variation in host plants (Heath and Tiffin 2007; Heath et al. 2010) and promote ecological coexistence of host genotypes (Parker 1999). Soils containing a mixture of symbiont strains could increase overall group fitness, by allowing greater potential for resource partitioning through increased assortative interactions with microbial partners. Variation in the identity or composition of symbiotic microbes could also have the opposite result, by enhancing competitive asymmetry between individuals in nonkin groups by, for example, favoring a subset of genotypes that are able to capitalize more resources from symbiotic partners compared to other genotypes (Wilkinson and Parker 1996).

We investigated whether plant competition between kin is stronger than competition between nonkin in an annual legume, *Medicago lupulina*, previously shown to have high selfing rates and strong genetic spatial structure due to passive seed dispersal (Yan et al. 2009), suggesting high frequency of interactions with closely related kin. Using plant lines from a single randomly sampled source population, we grew full siblings in kin groups or nonkin groups. We also examined the role of belowground nitrogen-fixing soil microbes in plant competition by growing plants in single and mixed inoculations of rhizobial partners. We predicted that mixed inoculations would increase the potential for niche partitioning by reducing overlap in resource competition between plant genotypes in nonkin groups.

Using an analytical approach developed in the productivity–diversity literature (Loreau and Hector 2001), we used mean individual fitness observed in kin groups to calculate null expectations for nonkin groups, under the null hypothesis that competitive dynamics between kin and nonkin do not differ. With this approach, we asked the following questions: (1) Does nonkin group fitness differ from expectations based on competitive dynamics in kin groups, and if so, what traits are potentially influencing competitive outcomes between individuals in a group? (2) Does the microbial community affect competitive dynamics among plant genotypes grown in kin or nonkin groups?

## Materials and Methods

### Study system

*Medicago lupulina* is naturalized Eurasian exotic annual (*Fabaceae*) growing in disturbed habitats, open fields, and roadsides throughout North America (Turkington and Cavers 1979). *Medicago lupulina* can outcross, but selfing is very high, 95.8% (Yan et al. 2009). Seeds disperse passively and populations exhibit strong spatial structure (Fst = 0.535 Yan et al. 2009). For our greenhouse experiment, we used 15 maternal plant families, randomly sampled from the largest *M. lupulina* population (approximately 40 m^2^) at the Koffler Scientific Reserve (Lat: 44°1′47.26″N, Long: 79°32′2.62″W; http://ksr.utoronto.ca/) as seed. Prior to the experiment, we allowed each maternal line to self for one generation in the greenhouse to equalize maternal and environmental effects; we refer to these maternal families as either lines or genotypes below. All experimental seeds were collected from a single individual parental plant per line. All lines were previously shown to exhibit significant broad-sense quantitative genetic variation in shoot and root biomass and flowering time (Simonsen et al. 2010).

*Medicago lupulina* forms mutualistic associations with several nitrogen-fixing species in the *Ensifer* clade, most commonly *Ensifer medicae* in southern Ontario (Prévost and Bromfield 2003). For our study, we used two *E. medicae* strains, RB7 and T2. Although T2 was originally isolated from *Melilotus alba*, *M. lupulina* also grew in proximity and represents a common host T2 is likely to encounter in old field and agricultural environments (Bromfield et al. 2010). Previous inoculation screenings showed that both strains form nitrogen-fixing nodules on *M. lupulina* (Simonsen and Stinchcombe, unpubl. data).

### Experimental design

We determined whether the outcome of competition differed in two environments: competition between kin (i.e. *,* kin groups composed of single genotypes) and competition between nonkin (i.e., multiple genotypes in nonkin groups). To increase the representation of nonkin interactions, we grew groups containing multiple individuals at the same density in either 2, 4, or 8 lines. We evaluated whether the rhizobial strain identity could modify the competitive interactions between genotypes by fully crossing all competition treatments with three rhizobia inoculation treatments: (1) RB7, (2) T2, and (3) 1:1 mixture of both rhizobia strains. For the kin groups, all 15 genotypes were replicated (*n*  = 3/rhizobia treatment combination, *n*_kintotal_ = 135). Each nonkin group at the specified family number was independently constructed by randomly sampling (without replacement) from the pool of 15 families (*n*  = 24/nonkin family number; *n*  = 8/rhizobia treatment, *n*_nonkintotal_ = 72), generating a random representative sample of all possible family compositions in each nonkin treatment (2, 4, and 8 families).

Prior to planting, we stratified all seeds at 4.0°C for 36 h and allowed radicles to grow for 24 h at 22°C. We planted 16 plants per pot (15.24 cm diameter ×25.4 cm height) to ensure seedling establishment and thinned to eight plants/pot 2 weeks following planting. In previous experiments, we found eight seedlings/pot was sufficient to reduce individual biomass compared to plants grown singly, indicating that the experimental density was sufficient to produce competition (Simonsen, A.K. and J.R. Stinchcombe, unpubl. data). Treatments were arranged in a randomized, blocked design in the greenhouse (2 replicates for each treatment combination/block, ×4 blocks). We grew both rhizobia strains in TY media (see Somasegaran and Hoben 1994) for 48 h and diluted both bacteria cultures to the same cell density (∼10^6^ cells/mL, OD_600_ = 0.1) and inoculated each seedling with 2 mL of culture evenly over the whole pot. We planted seedlings on May 27 and exposed plants to natural light–dark cycles throughout the growing season, with no supplemental artificial light.

### Plant traits measured

We tracked the identity of each individual. During the experiment, we scored height 30 days (early season) and 60 days (mid-season) after seedling establishment and flowering time. We terminated the experiment after 135 days to coincide with natural frost timing that ended the growth season and destructively harvested all plants. We measured dry aboveground shoot biomass and total seed and flower number for each individual. We estimated the probability of flowering (fraction of plants that reached flowering phase) and root biomass at the pot level, as disentangling roots between individuals proved impossible. Finally, we calculated the variance in shoot biomass among individuals in a pot, as a measure of inequality within a group.

### Testing for quantitative genetic variation in kin groups

We first tested for broad-sense genetic variation using data from kin groups to confirm that all traits were genetically variable. For seed data, we used a generalized linear mixed model with an overdispersed Poisson distribution to correct for multiple nonreproducing individuals. For flowering time, shoot biomass, and height, we used mixed models with standard Gaussian distributions. All fitness and trait data were analyzed at the individual level (with the exception of root biomass at the pot level), where block, harvest date, and rhizobia treatment were included as fixed effects, and random effects included pot, line, and line*rhizobia strain interaction. We tested for significant genetic variation using log-likelihood ratio tests between full models and reduced models without the random term of interest, that is, line and line*rhizobia strain (c.f. Littell et al. 1996).

### Quantifying kin and nonkin competition

A common method for measuring the outcome of competition between kin and nonkin is to compare the trait on focal individuals in either kin or nonkin environments (e.g., Cheplick and Kane 2004; Masclaux et al. 2010). While this analysis makes standard statistical procedures easier to implement, it requires very large samples sizes to make generalizable inferences across many genotypes in multiple nonkin environments. Instead, we grew nonkin groups composed of random assemblages of the available genotypes. This means that not all possible combinations of genotypes were present, but the mixtures were representative of the possible combinations. Masclaux et al. (2010) suggested that the presence of a few competitively dominant genotypes in the pool could bias the means of mixtures. In other words, the composition of a mixture is confounded with its diversity (in this case one vs. multiple genotypes). The issue identified by Masclaux et al. (2010) is identical to a problem found in biodiversity–ecosystem functioning studies, sometimes referred to as the “sampling effect”.

We utilized standard analytical techniques initially developed to study intra- and interspecific competition among plant species (Loreau and Hector 2001), which is designed to deal with this sampling effect issue, called Relative Yield Total (RYT), or “yielding”. Yielding, ΔY_fit_, evaluates the null hypothesis that the fitness consequences of competing individuals in kin and nonkin groups are equal, and is calculated as the difference between the observed total fitness (Yo_*i*_) and the expected total fitness (Ye_*i*_) of a nonkin group:


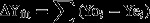
(1)

where *i* refers to the genotype in a mixture. The expected fitness (Ye_*i*_) is the null expectation that the fitness of a given genotype *i* in a nonkin group should be equal to its expected fitness based on how it grows with kin (calculated using mean fitness values of the genotype in kin groups). Equation 1 expresses a null model that genotypes grown in nonkin groups behave in the same way as when grown in kin groups. ΔY_fit_ gives a single quantitative measure of the difference in competition between kin and nonkin groups, while controlling for sampling effects that can obscure comparisons among nonkin group treatments (c.f. “sampling effects” in Loreau and Hector 2001). Positive yielding values indicate that plant groups, on average, attain higher group fitness when grown among nonkin and implies that competition between nonkin is, on average, weaker than competition between kin. Negative yielding values indicate that plant groups attain higher group fitness when grown among kin and that competition among nonkin is (on average) stronger.

We calculated ΔY_trait_ for all other traits (*i.e.,* shoot size, flowering time, root size, height) that we hypothesized could cause differences in competition between kin and nonkin groups. We calculated observed height, shoot biomass, and root biomass in nonkin groups using the same method as the seed data by summing individual trait data within a pot. We calculated the observed (i.e., Yo_i_) flowering time in nonkin groups as the mean date of flowering for plants within a pot. As with the seed count data, we calculated the expected trait values (i.e., Ye_i_) in nonkin groups using the mean of individual trait values for a given genotype in kin groups.

We calculated deviation from the null expectation of variance in plant size, ΔYvar_size_, in an analogous manner to ΔY_fit_. The expected variance, Ye_var_ in a given nonkin group, was calculated using the law of total variation:


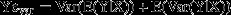
(2)

where Y is shoot size and X refers to a genotype within nonkin groups (“E” is a standard notation to denote an expected value – the mean – in probability theory). Verbally, the expected variance, Ye_var_ of a nonkin group, is calculated as the variance of the means of each genotype found in the mixture, plus the mean of the within line variances for each line in the mixture (where the within line variance is estimated from the kin group treatment). We elected to use variance in size (defined by Eqn. 2) rather than the Gini coefficient (Damgaard and Weiner 2000), because calculating the expected variance of a mixture, Ye_var_ as a function of statistical properties of its constitutive variables, is analytically straightforward from the rules of probability.

### Analysis of mean yielding values

We analyzed yielding values calculated at the pot level. We first used standard one sample *t* -tests (proc *t* -test, *h*  = 0, SAS v.9.2, SAS Institute Inc., Cary, NC, USA) to determine whether ΔY_fit_, ΔY_trait_, or ΔYvar_size_ values in nonkin groups differed from the expected values in kin groups – that is, whether there was evidence for differences in kin/nonkin competition. “We estimated q-values to account for multiple t-tests (c.f. Storey 2002).” Second, we used general linear models (proc glm, SAS v.9.2) to determine whether ΔY_fit_ or any ΔY_trait_ differed between rhizobia strain or genotype number, where block was a fixed effect. Finally, we regressed ΔY_fit_ against all other ΔY_trait_ values to determine what traits covaried with yielding in fitness.

### Trait contribution to fitness

We performed an additional analysis to determine how individual trait values predicted individual fitness (seed number) within kin and nonkin groups separately. We used a generalized mixed model approach (proc glimmix, SAS v.9.2), modeling seed production using an overdispersed Poisson distribution, including block as a fixed effect, date of harvest as a covariate, and pot and line as random effects. We included individual trait data (height, shoot, and flowering time) and their second-order terms (quadratic and interaction terms) as covariates in our model. Because we explicitly wanted to test how individual traits (height, shoot biomass, flowering time) contributed to seed production (and seed yielding differences), the analysis only included individuals that produced at least one seed.

## Results

### Genetic variation for traits in kin groups

Our initial analyses confirmed that all 15 lines exhibited significant genetic variation for nearly all measured traits in kin groups: early height (*χ* ^2^ = 27.9, *P*  < 0.001), mid-season height (*χ* ^2^ = 47.6, *P*  < 0.001), growth rate (*χ* ^2^ = 45.5, *P*  < 0.001), seed production (*χ* ^2^ = 54.9, *P*  < 0.001), aboveground biomass (*χ* ^2^ = 13.3, *P*  < 0.001), and date of first flower (*χ* ^2^ = 72.6, *P*  < 0.001) all showed significant genetic variation among lines, although root biomass (at the pot level, *χ* ^2^ = 1.2, *P*  = 0.14) did not. In addition, we only found marginal significance for seed production (*χ* ^2^ = 1.5, *P*  = 0.11).

### Patterns of yielding in seed production

Initial analysis showed no differential response in yielding values for fitness or any traits among differing family richness levels in nonkin groups. For the remainder of the analysis, we collapsed comparisons to two groups: kin versus nonkin.

Most genotypes showed consistent underyielding in seed number in nonkin groups (ΔY_fit_ = −128.2, *t*_(71)_ = −2.95, *P*  = 0.0043; Table1), which indicates that competition among genotypes in nonkin groups is, on average, stronger than competition within kin groups. Consistent with the underyielding result, individual seed production is lower in nonkin groups as well (*μ*_kin_ 89.80 ± 4.76 seeds/individual; *μ*_nonkin_ 65.77 ± 4.83 seeds/individual). However, we found no deviation from the expected number of plants that flowered in nonkin groups (ΔY_trait_ = −0.0532, *t*_(71)_ = −0.25, *P*  = 0.8062), suggesting that fitness underyielding was not due to variation in survival to flowering. We also found no substantial rank change in the mean individual seed production of genotypes between kin and nonkin treatments (*r*  = 0.98608, *P*  < 0.0001; Fig. S2). Differing number of genotypes in nonkin groups had no significant main effect on the degree of underyielding in fitness (*F*_1,63_ = 0.06 *P*  = 0.803; Fig. S1). As the number of genotypes within nonkin groups had no significant effect on fitness or any other traits, the remainder of our analysis is restricted to comparisons between two groups: kin and nonkin.

**Table 1:** Overall yielding values and standard error (SE) for fitness (ΔY_Fit_) and morphological traits (ΔY_trait_, ΔYvar_size_) across all nonkin groups for all rhizobia strain treatments

Trait	ΔY_Fit_ or ΔY_trait_	SE	*t*	*P*	*Q*
Fitness (seed#)	−128.2000	43.4499	−2.95	0.0043	0.0143
Fitness (fruit#)	−17.6045	4.7653	−3.69	0.0004	0.0020
Early height (cm)	−2.6227	1.1319	−2.32	0.0234	0.0585
Mid-height (cm)	−3.4038	3.0219	−1.13	0.2638	0.3769
Flowering time (days)	6.6039	3.5127	1.88	0.0642	0.1284
Shoot (g)	−0.3864	0.3408	−0.25	0.8062	0.8062
Root (g)	−0.3046	0.8340	−0.37	0.7160	0.8062
Shoot:root	0.0475	0.0302	1.58	0.1195	0.1992
Proportion flowered	−0.0532	0.2160	−0.25	0.8062	0.8062
Inequality in shoot biomass	1.1071	0.0902	12.25	<0.0001	<0.0010

Yielding values are calculated as the deviation from the expected trait value measured in kin groups. Proportion flowered are the number of individuals that flowered in a pot. *T* -tests indicate significant overall deviations from the expected yield. For all traits, *N*  = 72 and df = 71, except flowering time (*N*  = 68, df = 67). Adjusted *P* -values (*q*) using false discovery rate are shown to account for multiple *t* -tests.

### Traits mediating underyielding in nonkin groups

Initial height, flowering, and variance in shoot size significantly differed from their expectations, with initial height being shorter, flowering time being later, and inequality in shoot size being larger in nonkin groups (*t*_(71)_ = −2.32 *P*  = 0.0234, *t*_(67)_ = 1.88 *P*  = 0.0642 and *t*_(71)_ = 5.59 *P*  < 0.0001 respectively; Table1). Yielding in mid-season height, final shoot, or root biomass could not explain seed underyielding, as none deviated from the expected trait value (Table1). However, plants in nonkin groups showed a nonsignificant trend for overyielding in shoot to root allocation, with nonkin groups allocating slightly more to shoot production (Table1; *t*_(71)_ = 1.58 *P*  = 0.1195). These results suggest that stronger competitive environments in nonkin groups translated into differing competitive dynamics between genotypes that were expressed very early in the growth phase. Because mid-season height, final shoot, and root biomass showed no difference from expected trait values, and plants tended to allocate more to shoots rather than roots at harvest, it further suggests that some form of compensatory growth occurred during the later stage of the experiment in nonkin groups.

While we found no difference in total group aboveground biomass between kin and nonkin groups (Table1), two additional results indicate that there was a shift in the distribution of total biomass among individuals growing in nonkin groups. First, based on across-environment genetic correlations in shoot biomass, we found that genotypes that were large in kin groups also tended to be large in nonkin groups (*r*  = 0.67944, *P*  = 0.0053), suggesting little rank change in size across kin and nonkin environments. Second, greater variance in shoot biomass was observed among individuals in nonkin groups (s.e._nonkin_ = 0.184 and s.e._kin_ = 0.063), and the increase in inequality in nonkin groups was greater than expected based on size inequality in kin groups (Table1). These analysis indicate that, while the overall rank in individual fitness and size remained between kin and nonkin groups, large genotypes became larger, while smaller genotypes became smaller, resulting in greater inequality between individuals in nonkin groups.

### Trait contribution to fitness

**Figure 1:**
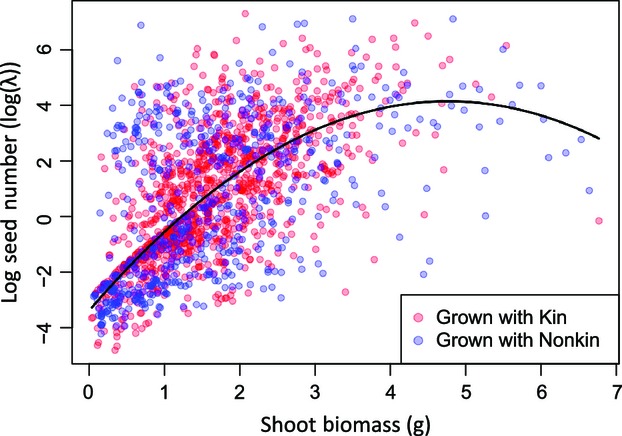
Fitness as predicted by biomass. Each data point represents fitness of an individual plant. The fitted curve is based on parameter estimates (*λ*) from a mixed overdispersed Poisson model, incorporating block as a fixed effect, shoot mass and flowering time as covariates and pot and plant family as random effects.

Analysis of ΔY_fit_ suggested several ΔY_trait_ that independently predicted the degree of underyielding in nonkin groups. Generally, nonkin groups that flowered later than expected, or showed the largest reduction in early height, under yielded the most in fitness (Table2). Genotypes with higher mean shoot biomass in kin environments also tended to either produce more seeds than expected, or underyield the least in seed number (Fig. S3), indicating that larger size provided some fitness advantage in nonkin groups. Our analysis of individual fitness and trait data showed that flowering time, early height, and shoot size were strong predictors of fitness (Table3). Height measured at mid-season (60 days) was not significant and removed from the model. However, we also found significant negative second-order terms in shoot size and flowering time (Table3) – shoot biomass and flowering time showed saturating relationships with seed production (Fig.1). The saturating relationship between shoot biomass and seed number suggests that, while plants gained some fitness advantage by suppressing their neighbors through larger size, the fitness advantage of neighbor suppression saturates when plants become larger. Follow-up analysis modeling how shoot biomass predicts fitness as a single trait showed that there was no significant difference in the magnitude of linearity (*F*_1,1577_ = 0.03, *P*  = 0.8553) or curvature (*F*_1,1580_ = 1.17, *P*  = 0.2796) between kin and nonkin groups.

**Table 2:** Yielding in fitness (seed number), ΔY_fit_, at the pot level regressed against yielding values for all trait values, ΔY_trait_ and ΔYvar_size_, and rhizobia strain treatments in a single ANCOVA model

ΔY_trait_	Estimate	SE	*F* value	df (num, den)	*P*
Early height (cm)	17.0262	4.6824	13.22	1,55	0.0006
Mid-height (cm)	1.9996	2.4119	0.69	1,55	0.4107
Flowering time (days)	−5.6125	1.1664	23.15	1,55	<0.0001
Shoot (g)	−53.8403	31.9642	2.84	1,55	0.0978
Root (g)	33.2818	18.1970	3.35	1,55	0.0728
Shoot:root	714.8700	464.6000	2.37	1,55	0.1296
Inequality in shoot biomass	47.0618	51.6352	0.83	1,55	0.3660
Strain treatment	–	–	2.83	2,55	0.0676

Estimates are the model parameter estimate from the ANCOVA showing the relationship between ΔY_fit_ and all other ΔY_trait_ trait values. Flowering time represents the date to first flower. Inclusion of the rhizobia treatment allows a test of whether strain treatments affected underyielding in fitness controlling for differences in trait morphology. SE is standard error.

**Table 3:** Multiple regression of seed production on phenotypic traits, at the individual level within kin and nonkin groups separately

	Estimate	SE	df (num, den)	*F* value	*P*
Kin Groups					
Shoot biomass (g)	0.3101	0.05772	1,488.6	28.86	<0.0001
Early height (cm)	0.1777	0.05382	1,480.3	10.91	0.0010
Flowering time (days)	−0.8081	0.05501	1,483.5	215.83	<0.0001
Shoot^*^Shoot biomass	−0.0997	0.02725	1,483.4	13.39	0.0003
Early height^*^ early height	−0.0661	0.02956	1,486.5	5.01	0.0257
Flowering time^*^Flowering time	−0.1177	0.03414	1,483.4	11.89	0.0006
Nonkin groups					
Shoot biomass (g)	0.1744	0.07944	1,248.1	4.82	0.0290
Early height (cm)	0.2054	0.08065	1,234.8	6.49	0.0012
Flowering time (days)	−0.6364	0.06943	1,237.9	84.01	<0.0001
Shoot^*^Shoot biomass	−0.0431	0.01968	1,242.0	4.79	0.0295
Early height^*^ early height	−0.0875	0.05242	1,239.8	2.78	0.0965
Flowering time^*^Flowering time	−0.1031	0.04453	1,211.1	5.36	0.0216
Early height^*^Flowering time	−0.1764	0.06287	1,222.0	7.88	0.0055

Seed number was modeled using an overdispersed Poisson distribution, with block as a fixed effect, harvesting date as a covariate, pot and plant family as random effects. Only individuals that produced at least one seed are included in this model. Flowering time indicates date of first flower. Traits were standardized to a mean = 0 and std = 1. All trait interactions were included in the model, but only significant trait interactions are shown. SE is standard error.

### Rhizobia strain differ in their effects on seed underyielding

**Figure 2:**
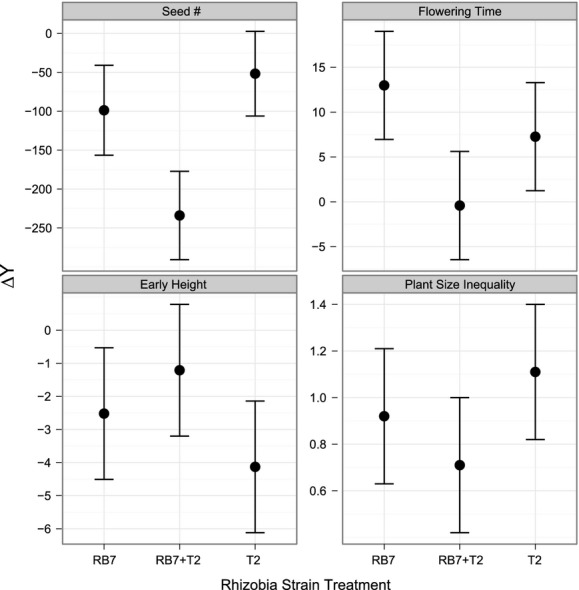
Yielding trait (early height, inequality in plant size, flowering time) and fitness values across strain treatments.

Mixed rhizobia treatments (T2 + RB7) showed the greatest underyielding in seed production, after accounting for all other ΔY_trait_ (Fig.2A; contrast test between single and mixed treatments: *F*_1,59_ = 4.96, *P*  = 0.0298; Table4). Higher underyielding in the mixed inoculation was driven by higher mean fitness in kin groups and slightly lower mean fitness in nonkin groups (*μ*_kin_ = 94.691 ± 9.230; *μ*_nonkin_ = 64.688 ± 8.572), compared to mean fitness in single inoculations between the two competition treatments (*μ*_kin_ = 87.356 ± 5.443; *μ*_nonkin_ = 66.307 ± 5.8404). However, early height underyielded and flowering time overyielded *the least* in the mixed rhizobia treatment (Fig.2B andC; Table4), indicating that reductions in early height and delays in flowering time could not explain the greater seed underyielding in mixed rhizobia treatments. Variance in aboveground shoot biomass also could not explain the underyielding pattern either, as mixed rhizobia treatments overyielded the least in shoot biomass inequality (Fig.2D). These results suggest that the effects of mixed rhizobia inoculations acted independent of all other yielding values of ΔY_trait_ and ΔYvar_size_ traits measured in this study to produce the overall underyielding effect in fitness.

**Table 4:** Comparison of yielding values for fitness and other plant traits in mixed [M] versus single [S] rhizobia strain inoculations

Traits	Mixed strain treatment [M] (ΔY_Fit_ -or- ΔY_trait_)	Single strain treatments [S] (ΔY_Fit_ -or- ΔY_trait_)	Direction of Differences
Fitness (seed#)	−**170.2000 ± 76.1647**	−**107.1000 ± 53.1998**	M>S
Fitness (fruit#)	−**24.3662 ± 8.2546**	−**14.2236 ± 5.8356**	M>S
Early height (cm)	−1.2141 ± 2.1289	−**3.3270 ± 1.3276**	M<S
Mid-height (cm)	−3.0402 ± 5.1418	−3.5856 ± 3.7712	M=S
Flowering time (days)	−0.4626 ± 10.7862	**10.5578 ± 1.8825**	M<S
Shoot (g)	−0.7622 ± 0.4564	−0.1984 ± 0.4582	M=S
Root (g)	0.4082 ± 1.5787	−0.6610 ± 0.9788	M=S
Shoot:root	0.0234 ± 0.0535	0.0596 ± 0.0368	M=S
Proportion Flowered	−0.3636 ± 0.3604	0.1020 ± 0.2691	M=S
Variance in shoot biomass	**1.0303 ± 0.1616**	**1.1454 ± 0.1093**	M=S

We compared yielding by performing contrast tests between [S] and [M]. Bold numbers indicate significant differences from zero, where *P*  < 0.05. Reported yielding values in the table are least-square means estimated during contrast test analysis.

## Discussion

Understanding how the strength of competition varies between kin and nonkin underpins several important conceptual questions in biology, including kin selection in evolutionary theory and niche partitioning in ecological theory. Competition between close relatives in neighboring plants has led to two major contrasting predictions: (1) niche-partitioning theory predicts that competition between close relatives will be stronger because close relatives are more likely to have overlapping niches and (2) kin selection theory predicts that competition between relatives will most likely be weaker because kin selection will reduce competition between close relatives. We found that competition between kin is weaker compared to competition between nonkin. Furthermore, we found that the composition of symbiotic nitrogen-fixing bacteria differentially affects plant competition in kin and nonkin groups. Specifically, a mixture of symbiotic nitrogen-fixing bacteria strains enhanced fitness, but only when host plants were grown among kin. Furthermore, our analysis was able to determine that depressed fitness in nonkin groups was due to competitive asymmetry in size acted independently from effects due to differences in rhizobia composition. Below, we discuss mechanisms leading to stronger competition in nonkin groups. In particular, we explain the role of Jensen's inequality in causing reduced fitness in nonkin groups. We also discuss implications of microbially mediated plant competition between kin and nonkin and potential explanations on how rhizobia communities containing a mixture of strains can increase plant fitness.

### Mechanisms of underyielding in fitness in nonkin groups

Plants, on average, experienced lower competition when grown beside their kin than in groups of nonkin. Our data are consistent with two explanations. The first explanation for a reduction in fitness in nonkin groups is due to delayed flowering time. The greater than expected delay in flowering time could be explained by an (unmeasured) allocation to root growth during early development among nonkin (inferred from reduced early height), which diverted resources to growth, potentially resulting in reduced future reproduction. Increased allocation to roots among nonkin has been interpreted as kin recognition (e.g., Dudley and File 2007), or (but not mutually exclusively) a “tragedy of commons” (e.g., Biernaskie 2011) – individuals selfishly increase allocation to roots to increase competitive ability at the expense of lowering total group fitness. However, differential root–shoot allocation could result from nonadaptive plastic responses to altered soil or root conditions.

While it is possible that plasticity in root to shoot biomass during early stages of growth may have occurred in our experiment and consequently delayed flowering onset, our study supports an alternative interpretation that requires neither kin-specific plastic responses nor kin recognition. Variation in size could be a mechanism explaining why plants have decreased individual fitness when grown among nonkin, and several pieces of evidence support this interpretation. First, we found no deviation from expected aboveground biomass in nonkin groups, but we did find higher than expected inequality in aboveground biomass (Table1). These results suggest that higher competition among nonkin exacerbated differences in aboveground shoot size between genotypes beyond their expected sizes, due to exploitative or interference competition (i.e., competition for light or soil space). When smaller genotypes experienced a reduction in size among larger nonkin, there was a proportional reduction in seed production. However, when larger, more competitive genotypes increased in size among nonkin, it did not follow that there was a proportional increase in seed production, because the relationship between size and seed production follows a curvi-linear relationship, showing diminishing fitness returns (Fig.1). Nonlinear relationships between size and fecundity are common in natural environments – one-third of species evaluated by Aarssen and Taylor (1992) – and could be generated by many potential causes, such as density dependent root competition, the length of the growing season, soil fertility or, if there is a minimum size for reproduction. Furthermore, reduced mean fitness in nonkin groups can be caused by nonlinear relationships between fitness and *any* trait associated with competitive ability (other than size), including unmeasured or undetected traits (e.g., rates of belowground resource acquisition).

Reduced fitness in nonkin groups can, in fact, be explained entirely by Jensen's inequality (Jensen 1906) in our experiment, without the need for kin recognition mechanisms or differential allocation responses. Jensen's inequality permeates many major patterns in ecology and provides predictive insight into interpreting means versus variances among treatments or groups (Bolnick et al. 2011). Jensen's inequality applies to situations where a response variable is predicted either by a decelerating or accelerating function, *f* (x) of an explanatory variable *x,* and the variance of the explanatory variable is greater than zero. Under these conditions, the predicted response evaluated at the mean, 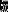, 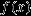 will not equal the mean of all the predicted values of the function, 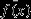, evaluated for all values of *x*. For decelerating functions, which we observed between size and seed production, even if the mean shoot biomass between kin and nonkin groups were the same (as was the case in our experiment), a higher variance (i.e., higher inequality) in size would depress the overall mean seed production in nonkin groups. Given higher inequality in size we observed in nonkin groups, it is therefore a mathematical expectation that groups composed of mixtures of genotypes will show reduced seed production. Interestingly, like Willson et al. (1987), we found lower early height in nonkin groups, which could also be driven by Jensen's inequality due to genetic differences in intrinsic growth rates (Łomnicki and Symonides 1990). It is also important to note that Jensen's inequality can also potentially explain higher fitness in nonkin groups, that is, due to accelerating functions between size and fitness when variance for size is higher in nonkin. Similarly, studies showing a lack of difference in fitness between kin and nonkin (e.g., Masclaux et al. 2010) may be due to a lack of nonlinearity between size or a relevant competitive trait and seed production.

File et al. (2012b) emphasize that kin selection does not explicitly require kin recognition mechanisms. Our study supports the view that competitive asymmetry can actually generate the same fitness patterns predicted by kin selection without requiring kin recognition traits or differential allocation (i.e., to roots and shoots). Given passive seed dispersal mechanisms and spatial aggregations of related individuals (Cheplick 1992), groups will often be of similar size or competitive ability if there is any quantitative genetic component to either of these traits. As quantitative genetic variation in these traits is commonly found in natural plant populations (e.g., Simonsen and Stinchcombe 2010; Donohue 2003; Johnson and Agrawal 2005; Crutsinger et al. 2006), it is difficult to exclude the hypothesis that reduced competition among sibling groups of plants is due to a by-product of genetic variation in size or competitive ability and in that regard provides a more parsimonious and generalizable explanation.

It may be possible with future work to develop analytical or statistical methods, or experimental designs, that can quantify and control for the possibility of Jensen's inequality affecting comparisons between kin and nonkin groups, in cases where it is likely to be an issue, although this is beyond the scope of the current study.

### Rhizobia communities modify competition

Legume hosts, *Medicago lupulina* included, will invariably encounter multiple rhizobia strains in natural soil (Denison 2000). If nonkin competed less strongly with each other because of niche-partitioning mechanisms, we would have expected nonkin groups to have either overyielding or the least degree of underyielding in mixed rhizobia treatments. However, we found that mixed inoculations actually increased seed underyielding in nonkin groups and acted independently of underyielding effects caused by competitive asymmetry brought about by differences in size inequality. The higher underyielding in mixed inoculations was primarily driven by higher group fitness in kin groups exposed to mixed inoculations. The effects of competitive asymmetry eliminate any positive fitness effects of mixed rhizobia cultures in nonkin groups.

Our experiment suggests two important implications regarding the effect of symbiotic microbes on plant competition between kin and nonkin groups: (1) Microbial symbiotic variation in the community can enhance fitness advantages of competing with kin rather than nonkin and (2) competitive asymmetry induced in nonkin groups can eliminate any differential fitness effects caused by the microbial community. Given the mixture of studies that either support kin selection, niche partitioning, or neither, it is clear that context matters in detecting either of these processes (e.g., Andalo et al. 2001; Lepik et al. 2012). For example, Ronsheim and Anderson (2001) found no detectable differences in overall group biomass between kin and nonkin groups unless mycorrhizal fungi were added to the soil mixture. These studies, as well as our own, demonstrate that the strength of kin competition will depend on the microbial environment in which plant competition is evaluated.

Agricultural studies have also found differences in plant yield as a result of co-inoculations (Medeot et al. 2010). One mechanism is through increased complementarity in nutrient availability. For example, mixed inoculation of *Rhizobium* strains with *Bacillus* strains on pigeon pea (*Cajanus cajan)* has been found to enhance plant growth, compared to single strain inoculations, because the *Rhizobium* strain enhances N-fixation, while *Bacillus* strains enhance iron availability (Rajendran et al. 2008). It is possible that the higher availability of strains in our experiment caused nutrient complementarity compared to single strain inoculations. However, mixed rhizobia inoculations have been shown to depress fitness (Heath and Tiffin 2007), or produce no effect at all (Somasegaran and Bohlool 1990). For example, Becker et al. (2012) found that increasing the diversity of *Pseudomonas fluorescens* strains in the soil caused increased antagonistic interactions between strains, resulting in decreased bacterial densities and root colonization. Lau and Lennon (2011) found that the microbial community composition can alter the strength of selection on ecologically important traits in plants. While further studies will be required to determine the strain–strain mechanisms that cause nonadditive effects on fitness, these studies as well as ours show that interactions between microbial partners can impact plant productivity and fitness and therefore have a role in influencing plant–plant competition.

## Conclusions

We have shown that reduced kin competition can be caused by the combined effects of genetic variation in size and saturating fitness functions with traits associated with competitive dominance (i.e., plant size), which can be entirely explained by Jensen's inequality. Our study also demonstrates that differences in microbial symbiont composition can alter competition between genotypes independent from the effects of competitive asymmetry. Our study identifies microbial community composition as a potential factor in explaining why some studies detect differences in fitness between kin and nonkin groups while others do not. For future studies examining competition between genotypes in mixtures, we recommend ruling out by-product effects that arise from Jensen's inequality before invoking or inferring niche-partitioning processes or adaptive processes that increase inclusive fitness.

## Data Accessibility

Data is available from Figshare repository (http://figshare.com/): http://dx.doi.org/10.6084/m9.figshare.1213852.

## Supporting Information

Figure S1 Yielding in fitness across nonkin treatments varying in plant family number.

Figure S2 Mean individual seed production for each plant family correlated across kin and nonkin environments.

Figure S3 Plant family that produced lower shoot biomass in kin groups showed the highest underyielding in seed production.
