# Supplementary material for: Reduced plant competition among kin can be explained by Jensen's inequality
Source: Ecol Evol. 2014 Nov 10;4(23):4454–66. doi: 10.1002/ece3.1312 (PMC4264895; doi:10.1002/ece3.1312)
Supplement: Figure S1 — Yielding in fitness across nonkin treatments varying in plant family number. [file ece30004-4454-sd1.docx]

**
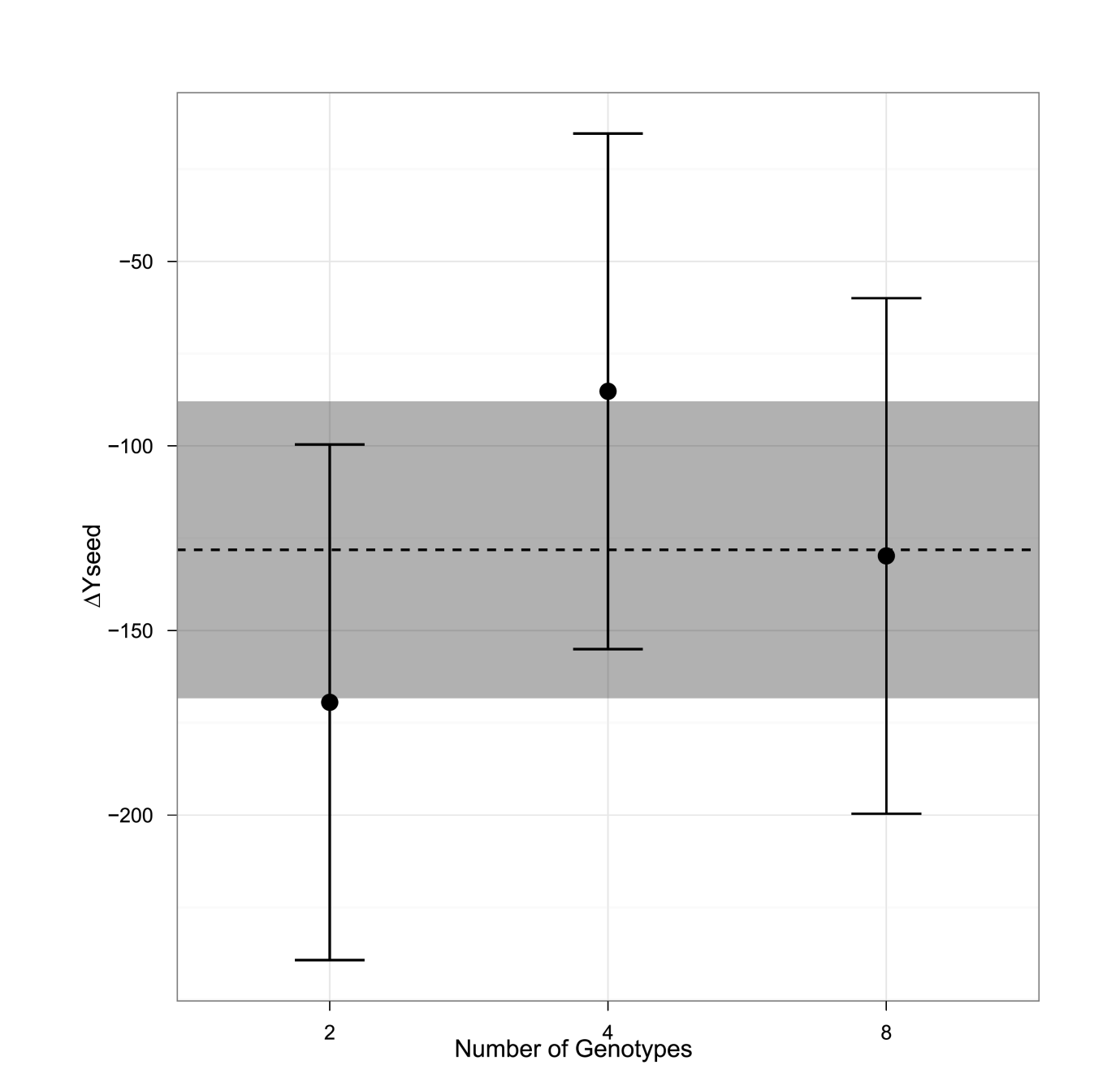
**

**Figure S1:** Yielding (±standard error) in fitness across non-kin treatments varying in plant family number. Negative values indicate lower than expected fitness in non-kin groups compared to fitness values predicted from kin groups. Dashed line indicates overall mean (±standard error indicated by shaded region) across all non-kin treatments.
