# Supplementary material for: Reduced plant competition among kin can be explained by Jensen's inequality
Source: Ecol Evol. 2014 Nov 10;4(23):4454–66. doi: 10.1002/ece3.1312 (PMC4264895; doi:10.1002/ece3.1312)
Supplement: Figure S2 — Mean individual seed production for each plant family correlated across kin and nonkin environments. [file ece30004-4454-sd2.docx]

**
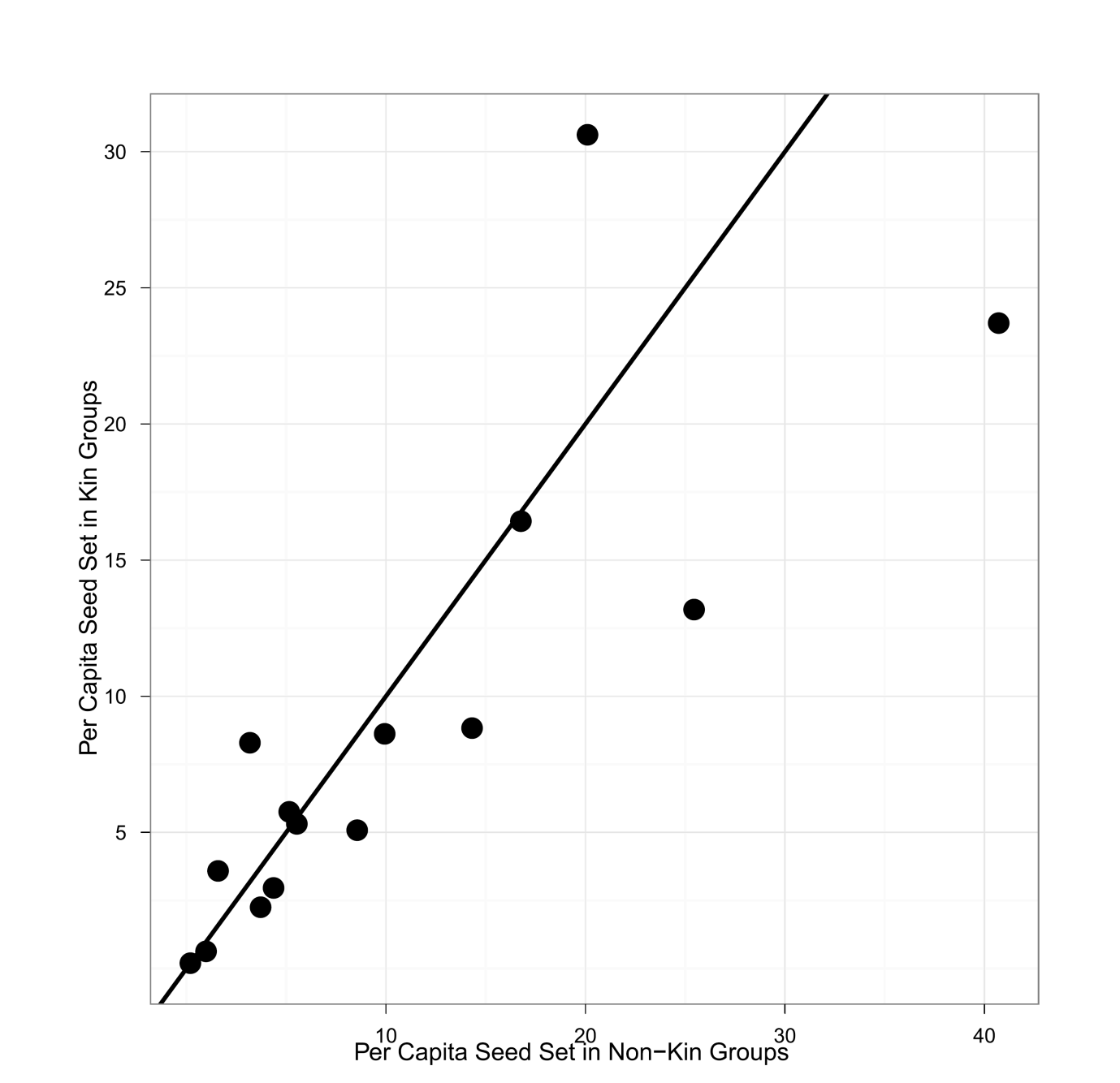
**

**Figure S2:** Mean individual seed production (seed number) for each plant genotype correlated across kin and non-kin environments. A 1:1 line is shown for comparison.
