# Supplementary material for: Reduced plant competition among kin can be explained by Jensen's inequality
Source: Ecol Evol. 2014 Nov 10;4(23):4454–66. doi: 10.1002/ece3.1312 (PMC4264895; doi:10.1002/ece3.1312)
Supplement: Figure S3 — Plant family that produced lower shoot biomass in kin groups showed the highest underyielding in seed production. [file ece30004-4454-sd3.docx]

**
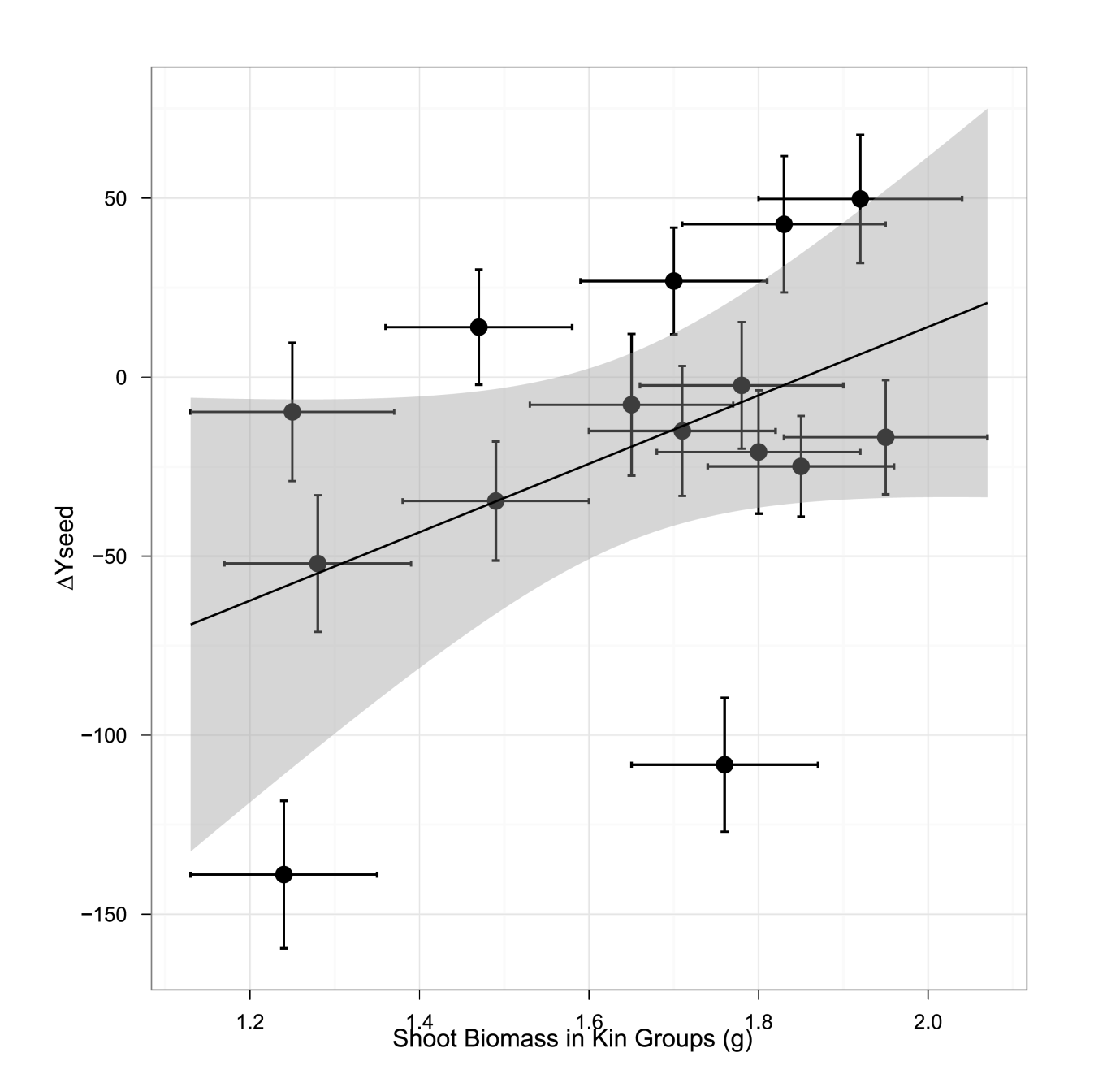
**

**Figure S3:** Plant families that produced lower shoot biomass in kin groups showed the highest underyielding in seed production. Each data point is a mean trait (±standard error) value for each plant family calculated at the individual level. Shading indicates 95% CI for fitted line.
